# Homeownership status and risk of food insecurity: examining the role of housing debt, housing expenditure and housing asset using a cross-sectional population-based survey of Canadian households

**DOI:** 10.1186/s12939-019-1114-z

**Published:** 2020-01-06

**Authors:** Andrée-Anne Fafard St-Germain, Valerie Tarasuk

**Affiliations:** 1 Manitoba Centre for Health Policy, Department of Community Health Sciences, University of Manitoba, Winnipeg, MB Canada; 2 Department of Nutritional Sciences, University of Toronto, Toronto, ON Canada

**Keywords:** Food insecurity, Housing tenure, Market renters, Mortgage, Housing wealth, Canada

## Abstract

**Background:**

Household food insecurity is a potent marker of material deprivation with adverse health consequences. Studies have repeatedly found a strong, independent relationship between owning a home and lower vulnerability to food insecurity in Canada and elsewhere, but the reasons for this relationship are poorly understood. We aimed to examine the influence of housing asset, housing debt and housing expenditure on the relationship between homeownership status and food insecurity in Canada.

**Methods:**

Cross-sectional data on food insecurity, housing tenure and expenditures, home value, income and sociodemographic characteristics were derived from the 2010 Survey of Household Spending, a population-based survey. Multivariable logistic regression models were conducted to estimate odds ratios of food insecurity among households of all incomes (*n* = 10,815) and those with lower incomes (*n* = 5547).

**Results:**

Food insecurity prevalence was highest among market renters (28.5%), followed by homeowners with a mortgage (11.6%) and mortgage-free homeowners (4.3%). Homeowners with a mortgage (OR: 0.51, 95% CI: 0.39–0.68) and those without a mortgage (OR: 0.23, 95% CI: 0.16–0.35) had substantially lower adjusted odds of food insecurity than market renters, and accounting for the burden of housing cost had minimal impact on the association. Mortgage-free homeowners had lower adjusted odds ratios of food insecurity compared to homeowners with a mortgage, but differences in the burden of housing cost fully accounted for the association. When stratifying homeowners based on presence of mortgage and housing asset level, the adjusted odds ratios of food insecurity for market renters were not significant when compared to mortgage holders with low housing asset. Mortgage-free owners with higher housing asset were least vulnerable to food insecurity (adjusted OR: 0.18, 95% CI: 0.11–0.27).

**Conclusions:**

Substantial disparities in food insecurity exist between households with different homeownership status and housing asset level. Housing policies that support homeownership while ensuring affordable mortgages may be important to mitigate food insecurity, but policy actions are required to address renters’ high vulnerability to food insecurity.

## Background

Household food insecurity is a serious public health concern in many affluent countries, including Canada [1–4]. Extensive evidence suggests that lacking adequate or secure access to food due to financial constraints is an important marker of material deprivation that contributes to health inequalities across the life cycle independently of other social determinants of health [4–9]. This highlights the need to understand the different economic determinants of food insecurity to inform the development of effective policy interventions.

Canada has no governmental intervention aimed explicitly at reducing food insecurity, but like many other countries, it has several social programs to mitigate experiences of economic hardships. Recent studies suggest food insecurity is sensitive to federal and provincial income security programs, including universal old-age pension [10], child benefits [11, 12], and social assistance benefits [13, 14]. These findings are consistent with a large body of research identifying household income has a robust predictor of food insecurity in Canada [12, 14–20] and elsewhere [21–26].

Housing policy is an integral part of Canada’s welfare state [27–29], but unlike existing income security programs, the focus of housing policy is not limited to vulnerable population subgroups. In tandem with programs subsidizing housing cost for a small proportion of vulnerable low-income renters, several policies are in place to promote asset accumulation through homeownership [28, 29], and these have been especially effective for higher-income households [30]. Homeowners represent approximately two thirds of all Canadian households [31], yet they comprise only one third of food insecure households [3]. Food insecurity is four times less prevalent among homeowners than renters [3, 20], and while this disparity appears to be largely driven by economic and sociodemographic differences between renters and homeowners [16], population-based research suggests that homeownership confers some protection against food insecurity [12, 14, 16–20]. This is not unique to Canada since studies from other affluent countries also found lower food insecurity risk among homeowners than renters [21–26, 32].

As an important asset [33, 34], homeownership may reduce food insecurity risk by facilitating access to credit in times of financial constraints [16, 22, 35]. To date, studies from Canada [12, 14, 16–20] and elsewhere [21–26] examined the relationship between homeownership and food insecurity by differentiating renters from homeowners but none distinguished owners with a mortgage from those without a mortgage. Yet, mortgage holders may be at greater risk of experiencing food insecurity due to the financial vulnerability associated with having to service a substantial debt [36]. Housing affordability problems, defined as housing costing 30% or more of before-tax income, affect only 6.6% of mortgage-free homeowners but 23.0% of homeowners with a mortgage and 40% of renters [31]. Mortgage and rent payments can represent a large recurrent expense that may not only reduce households’ ability to afford food [37] but also their ability to save and buffer unexpected financial shocks [22, 23]. Little is known about the mechanisms underlying the disparities in food insecurity between households that rent, own with a mortgage and own mortgage-free, but investigating whether the housing asset per se and lower housing cost burden contribute to homeowners’ lower vulnerability to food insecurity would provide important insights on the role of housing policy in mitigating food insecurity.

Drawing on a unique Canadian population-based survey, this study aims to expand current understandings of the protective effect of homeownership by examining the influence of housing asset, housing debt and housing expenditure on the relationship between homeownership status and vulnerability to food insecurity.

## Methods

### Data and study sample

This study used data from the 2010 Survey of Household Spending (SHS), a cross-sectional survey representative of the population living in the ten provinces, except for individuals living in institutions, in military camps or on First Nations reserves [38]. These exclusions constitute approximately 2 % of the population in the provinces.

Data were collected during an in-person interview with the head of household [38]. For most households, detailed information on income for the year prior to the survey was retrieved from income tax records; otherwise, this information was collected during the interview. Missing values for the income and expenditure variables were imputed by Statistics Canada using the nearest neighbor imputation method, while the other variables included a missing category when applicable. A total of 13,075 households were interviewed [38], but the analytic sample for this study included one-person household and single census families that were market renters or homeowners with no missing data on food insecurity or highest level of education in the household, and with a total after-tax income equal to or larger than housing expenditure (*n* = 10,815). The focus on one-person household and single census families with no other persons increased the likelihood that the income and housing expenditure represented shared resources and costs. Renters paying a reduced rent (e.g., received rent for free or a subsidy from government, employers, landlords or family members) were excluded for analytical and conceptual reasons; as a group, these households had little variation in their housing cost burden, preventing examinations of whether housing cost burden explains difference in the risk of food insecurity among these households relative to renters paying market rent and homeowners. In addition, the heterogeneity in the reasons for reduced rent limits the interpretation of the vulnerability of these households to food insecurity in relation to their housing circumstances. Previous research focused on households paying reduced rent because of government housing subsidy documented high rates of food insecurity among these [15, 39, 40], which likely reflect the selection of highly vulnerable, low-income households into social housing programs in Canada [40, 41]. By including only renters paying market rent (market renters), the current analyses focus on the majority of renting Canadian households [31].

### Food insecurity outcome

Food insecurity was measured with the 18-item Household Food Security Survey Module (HFSSM), a validated questionnaire used for national monitoring in Canada and the US [3, 42, 43]. The HFSSM is an experience-based scale that measures food access problems caused by a lack of money. The scale refers to the past 12 months and differentiates the experiences of children and adults within the household. Due to an error in the administration of the 8 questions specific to children during the interview of the 2010 SHS, most households with children had missing data on these. Thus, this study used the 10 items specific to adults to determine food insecurity status. Responses were coded as affirmative based on Health Canada’s protocol [42]. Households with one or more affirmative answers were considered food insecure [3]. Health Canada conventionally defines food insecurity as two or more affirmative answers to the 10 items [42], but recent evidence suggests that any affirmative item is indicative of marginal food insecurity associated with unique socioeconomic profiles [14, 20] and poorer health outcomes [6, 7, 9].

### Housing variables

Homeownership status was a three-category variable differentiating market renters, homeowners with a mortgage and mortgage-free homeowners. To examine the influence of housing debt and expenditure, two measures of housing cost burden were created. The proportion of after-tax income allocated to housing (‘housing-to-income ratio’) represents a relative measure of housing cost burden and is consistent with conventional indicators of housing affordability used in Canada [31] and elsewhere [44]. The income left after paying for housing (‘after-housing income’) represents an absolute indicator of housing cost burden and captures housing-induced shortfall in income [45]. For market renters, housing expenditure was the sum of annual expenditures on rent, utilities (i.e. heat, electricity, water) and tenant’s home insurance, while for homeowners, it included annual expenditures on mortgage, mortgage insurance, utilities, owner’s home insurance, and property tax. The expenditure component most strongly correlated with total housing expenditure was rent among market renters (correlation *r* = 0.98), mortgage among mortgage holders (*r* = 0.96), and property tax among mortgage-free homeowners (*r* = 0.76) (Additional file 1: Table S1, S2 and S3). Households with an after-tax income smaller than housing expenditure were excluded because they were outliers with negative after-housing income and housing-to-income ratio greater than one. Housing expenditure and after-tax income were adjusted for economies of scale using the square root of household size [46].

To examine the influence of housing asset, a five-category variable was created by combining information on homeownership status and on whether homeowners had low or higher housing asset (≤$120,000 or > $120,000). In the absence of a standardized threshold to characterize housing asset level, low housing asset was defined as owning a home with a value in the lowest decile of home values in the sample (≤$120,000); this threshold appeared to identify homeowners with greater vulnerability to food insecurity (Additional file 1: Fig. S1). Homeowners with a home value in the second to tenth deciles were classified as having higher housing asset. Estimated home value was self-reported at the time of the interview and represented the amount homeowners would expect to receive if they were to sell their house [38].

### Covariates

The selection of control variables was informed by research identifying sociodemographic and economic characteristics associated with food insecurity [12, 14–22] and homeownership status [30, 34, 47] that could confound the relationships of interest. Given that food insecurity is measured at the level of the household, the variables were selected to represent household- rather than individual-level concepts.

The social epidemiology of food insecurity in Canada is well-established [12, 14, 16–20]. Previous population-based studies have repeatedly identified household income, main income source, education, household structure and composition, ethnicity, indigeneity and province or region of residence as independent risk factors for food insecurity [12, 14, 16–20]. Similar economic and sociodemographic characteristics have been shown to be associated with the propensity to own a home among Canadian households [30, 34, 47]. Analyses from Canada and the US also suggest that presence of a household member with disability or chronic health conditions is associated with greater vulnerability to food insecurity [15, 17, 21] and lower probability of owning a home [34]. Research indicates that life stage is an important determinant of homeownership and asset accumulation among Canadian households, with later stages typically associated with higher likelihood to own a home and greater overall assets [30, 33, 34, 47, 48]. Although the concept of life stage has not been explicitly examined in relation to food insecurity in Canada, population-based studies have shown that households primarily reliant on senior’s income are less vulnerable to food insecurity [12, 14, 16–18, 20], while families with children below the age of 18 tend to be more vulnerable [12, 14, 16–20]. Some studies have also documented higher risk of food insecurity among younger adult respondents [16, 17].

Based on the data available in the 2010 SHS and potential confounders identified in the literature, the control variables included in the analyses were: household structure, number of children below the age of 18, highest level of education achieved by the head of household or spouse, age of head of household, main source of household income, proxy indicator for presence of a household member with disability, region of residence and population centre size. In some of the analyses, it was possible to include after-tax income adjusted for household size as a continuous variable. However, due to multicollinearity between after-tax income and measures of housing cost burden, some of the analyses could not directly control for income. Thus, the analyses were first conducted among households of all income levels and then were repeated among lower-income households to minimize the confounding of income.

### Statistical analyses

Food insecurity prevalence by homeownership status and housing asset level was estimated for the whole sample and lower-income subsample, which included households with an adjusted after-tax income below or equal to the median ($37,417). Means and proportions were used to describe the sample by food insecurity status. Univariable and multivariable logistic regression models were conducted to predict the odds ratios of food insecurity in the whole sample and lower-income subsample. Since household income is the most commonly used measure of economic resources in analyses of food insecurity, a multivariable model with the three-category variable describing homeownership status, after-tax income and the other covariates variables was performed for comparison purposes. Then, after-housing income and housing-to-income ratio were included as continuous variables in separate models that included the covariates to examine the influence of housing expenditure and debt on the relationship between homeownership status and food insecurity. To investigate the influence of housing asset, the five-category variable that combined homeownership status with housing asset level was used to predict the odds ratios of food insecurity, while controlling for after-tax income and other covariates. As a sensitivity analysis, multivariable logistic regression models were conducted among homeowners to examine further how their vulnerability to food insecurity differed based on presence of mortgage and housing asset level. Sampling weights for the survey were used to obtain population-based estimates, and the 1000 bootstrap weights provided by Statistics Canada were used to account for the complex survey design in the estimation of standard errors and 95% confidence intervals. All analyses were conducted using Stata 15.

## Results

**Fig. 1:**
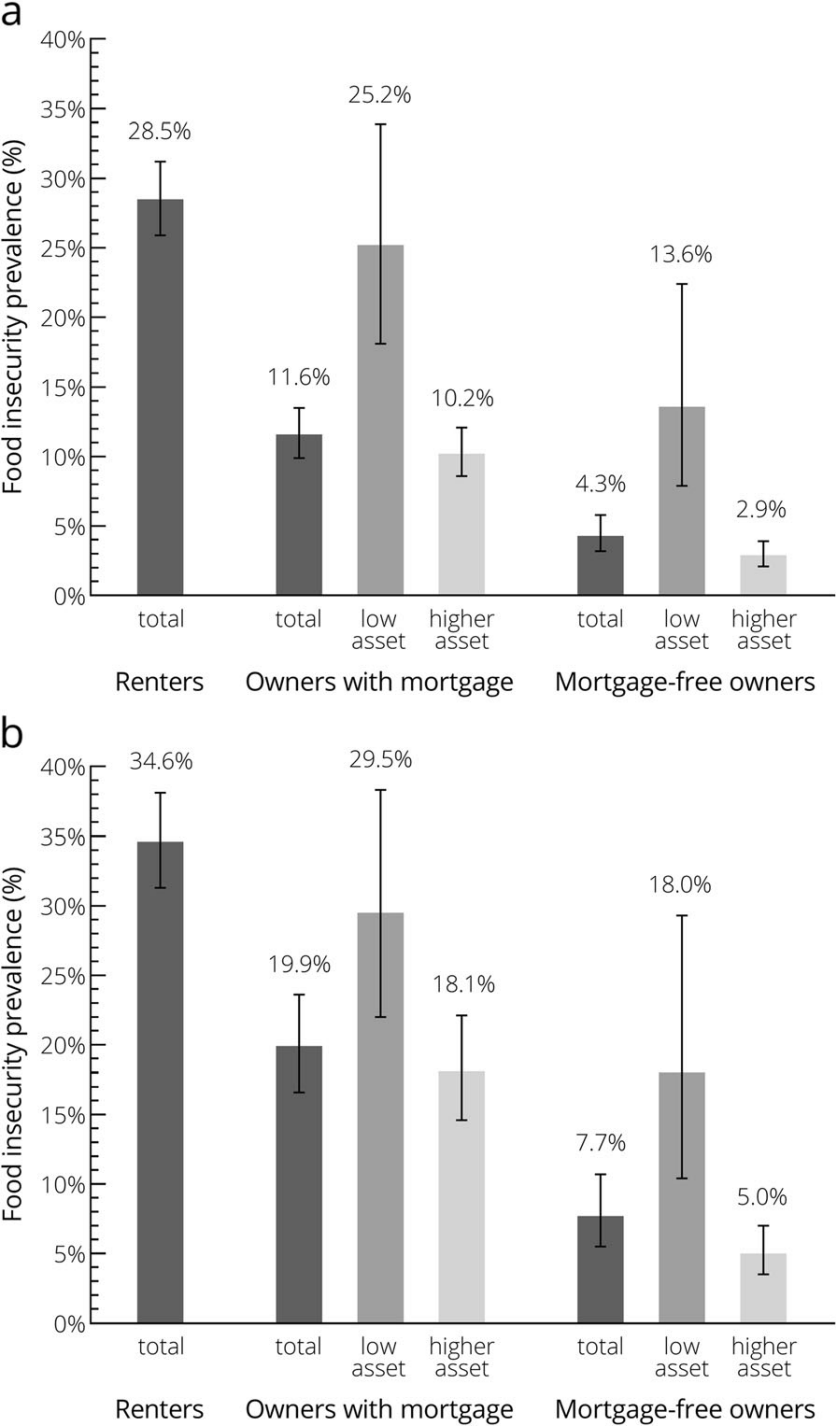
Food insecurity prevalence by homeownership status and housing asset level* among all households (**a**) and lower-income households (**b**). The error bars represent the 95%CI for the prevalence estimates. *Low housing asset defined as home value ≤$120,000, representing the lowest decile of home value; higher housing asset defined as home value >$120,000.

Food insecurity was most prevalent among renters (28.5%), followed by owners with a mortgage (11.6%) and mortgage-free owners (4.3%) (Fig. 1a). When stratifying homeowners based on housing asset level, the prevalence was highest among mortgage holders with low housing asset (25.2%) and lowest among mortgage-free owners with higher housing asset (2.9%), while the prevalence was mid-range for mortgage holders with higher housing asset (10.2%) and mortgage-free owners with low housing asset (13.6%). The prevalence estimates increased slightly when focusing on lower-income households, but the pattern across homeownership status and housing asset level was similar to that observed for the whole sample (Fig. 1b).

Food secure and food insecure households had different sociodemographic and economic profiles (Table 1). Food insecure households had, on average, lower after-tax income, housing expenditure and after-housing income, but higher housing-to-income ratio than their food secure counterparts.

**Table 1:** Descriptive statistics by household food insecurity status

Characteristics	All	Food secure	Food insecure	P value*
	n = 10,815	*n* = 9451	*n* = 1364	
Homeownership status, %				< 0.0001
Renters	26.8	22.1	56.4	
Owners with mortgage				
Low housing asset (≤$120,000)	3.4	2.9	6.2^§^	
Higher housing asset (>$120,000)	34.3	35.6	26.0	
Mortgage-free owners				
Low housing asset (≤$120,000)	4.7	4.7	4.7^§^	
Higher housing asset (>$120,000)	30.9	34.7	6.6	
After-tax income†, mean (SD)	$43,506 (33136)	$46,143 (34453)	$26,606 (16036)	< 0.0001
Housing expenditure†, mean (SD)	$8584 (5498)	$8658 (5699)	$8108 (4063)	0.014
After-housing income†, mean (SD)	$34,922 (32256)	$37,485 (33595)	$18,498 (14879)	< 0.0001
Housing-to-income ratio, mean (SD)	25.0% (17.2)	23.2% (16.0)	36.8% (19.5)	< 0.0001
Age of head of household, %				< 0.0001
< 30	8.6	7.6	14.9	
30 to < 40	17.3	16.4	23.0	
40 to < 50	21.8	20.7	28.8	
50 to < 60	21.1	21.6	17.9	
60 to < 70	15.9	17.1	8.7	
≥ 70	15.3	16.6	6.8	
Household structure, %				< 0.0001
Unattached, living alone	28.1	26.7	37.3	
Couple, living alone	32.2	34.9	14.8	
Couple with children‡	33.0	32.9	33.3	
Lone parent‡	6.8	5.5	14.6	
Number of children < 18, %				< 0.0001
0	72.1	73.6	62.6	
1	11.4	10.8	14.9	
2 or more	16.5	15.6	22.5	
Household member with disability¶, %				< 0.0001
Yes	21.2	19.5	32.3	
No	78.8	80.5	67.7	
Household education (highest level), %				< 0.0001
High school diploma or less	29.6	27.2	45.3	
Post-secondary	40.1	39.7	42.5	
University degree	30.3	33.1	12.2	
Main source of income, %				< 0.0001
Wages/salaries or self-employment	67.1	68.2	60.0	
EI or workers’ compensation	2.4	1.5	8.5^§^	
Seniors’ benefits or pension	23.4	25.1	12.3	
Social assistance	2.3	0.9^§^	11.1	
Other sources	4.9	4.3	8.1	
Region of residence, %				0.003
Atlantic	7.5	7.3	9.0	
Quebec	26.1	26.4	24.4	
Ontario	36.4	35.6	41.7	
Prairies	17.4	17.8	14.7	
British Columbia	12.6	12.9	10.4	
Population centre size, %				0.031
Rural (< 1000)	11.9	12.2	9.7	
Small (1000 to < 100,000)	20.9	20.2	25.5	
Medium (100,000 to < 500,000)	17.4	17.3	18.0	
Large (≥500,000)	49.9	50.3	46.8	

EI, Employment insurance

Column percentages may not add to 100% due to rounding

* *p value*s based on the Rao-Scott F adjusted chi-square statistic for categorical variables and the adjusted Wald test for differences in the means of continuous variables

† Adjusted for household size by dividing by the square root of household size

‡The presence children in these households reflects the familial relationship between the members of the household, and the children may be of all ages

¶ Presence of household member with disability based on households reporting that one member’s amount or kind of activity at home, at work, at school or in other activities was sometimes or often reduced by a physical/mental condition or health problem [38]

§ Coefficient of variation greater than 16.6%, indicating that the estimate is associated with high sampling variability

Compared to renters, the unadjusted odds of food insecurity were smaller among owners with a mortgage (0.33, 95% CI: 0.26–0.41) and mortgage-free owners (0.11, 95% CI: 0.08–0.16) (Table 2). Adjusting for the covariates and after-tax income led to weaker but still significantly lower odds ratios for both types of homeowner; compared to renters, owners with and without a mortgage had 49% (0.51, 95% CI: 0.39–0.68) and 77% (0.23, 95% CI: 0.16–0.35) lower adjusted odds of food insecurity, respectively. The odds ratios for mortgage-free owners were practically identical whether the regression model included after-tax income (0.23, 95% CI: 0.16–0.35), after-housing income (0.26, 95% CI: 0.17–0.39) or housing-to-income ratio (0.25, 95% CI: 0.17–0.39). In contrast, the lower odds ratios for owners with a mortgage compared to renters tended to be stronger (further away from 1) when the model included after-housing income (0.43, 95% CI: 0.33–0.56) or housing-to-income ratio (0.31, 95% CI: 0.24–0.41). Overall, these results suggest that differences in the burden of housing cost contributed minimally to the disparities in food insecurity between renters and both types of homeowner.

**Table 2:** Odds ratios of household food insecurity by homeownership status among households of all incomes (*n* = 10,815)

Independent variables	Unadjusted model	Covariates* + after-tax income	Covariates* + after-housing income	Covariates* + housing-to-income ratio
	OR (95% CI)	aOR (95% CI)	aOR (95% CI)	aOR (95% CI)
Homeownership status				
Renters	1.00	1.00	1.00	1.00
Owners with mortgage	**0.33 (0.26–0.41)**^a^	**0.51 (0.39–0.68)**^a^	**0.43 (0.33–0.56)**^a^	**0.31 (0.24–0.41)**^a^
Mortgage-free owners	**0.11 (0.08–0.16)**^b^	**0.23 (0.16–0.35)**^b^	**0.26 (0.17–0.39)**^b^	**0.25 (0.17–0.39)**^a^
After-tax income†	–	**0.96 (0.95–0.97)**	–	–
After-housing income†	–	–	**0.96 (0.95–0.97)**	–
Housing-to-income ratio†	–	–	–	**1.13 (1.09–1.17)**

aOR adjusted odds ratios, OR unadjusted odds ratios, CI confidence interval

Note: The logistic regression models used sampling weights to obtain population-based OR and aOR; the 95% CI are calculated using bootstrapped standard errors estimated with 1000 bootstrap weights provided by Statistics Canada to account for the complex survey design. Odds ratios in bold are significantly different from 1.00 (*p values* < 0.05)

^a, b^ Based on comparisons of odds ratios from the same regression model (same column), odds ratios with different superscripts differ significantly from each other (*p values* < 0.05), while odds ratios with the same superscript do not differ (*p values* ≥ 0.05). See Additional file 1: Table S4 for the odds ratios for mortgage-free owners versus owners with a mortgage

* Covariates include household type, number of children < 18 years of age, presence of household member with disability, age of head of household, household education, main income source, region of residence and population centre size

† Odds ratios for $1000 increase in after-tax or after-housing income and for 5% increase in housing-to-income ratio

The comparison of owners without a mortgage to those with a mortgage showed that the former had lower unadjusted odds of food insecurity (Table 2 and Additional file 1: Table S4). Even after adjusting for covariates and after-tax income, mortgage-free owners still had 55% lower odds of food insecurity than owners with a mortgage (0.45, 95% CI: 0.30–0.68) (Additional file 1: Table S4). The adjusted odds ratio tended to be weaker (closer to 1) when including after-housing income in the model (0.61, 95% CI: 0.41–0.90), and was non-significant when including housing-to-income ratio (0.81, 95% CI: 0.53–1.25). These results indicate that differences in the burden of housing cost contributed to the disparities in vulnerability to food insecurity between owners with and without a mortgage.

Limiting the analyses to lower-income households had minimal impact on the results and their interpretations (Table 3). After adjusting for covariates and after-tax income, the odds of food insecurity among owners with and without a mortgage were, respectively, 47% (0.53, 95% CI: 0.38–0.74) and 74% (0.26, 95% CI: 0.16–0.41) lower that the ones of renters. The adjusted odds ratios for mortgage-free owners remained similar whether the model included after-housing income (0.31, 95% CI: 0.19–0.50) or housing-to-income ratio (0.31, 95% CI: 0.19–0.50), while the adjusted odds ratios for owners with a mortgage tended to increase in strength (further away from 1) when including after-housing income (0.41, 95% CI: 0.30–0.56) or housing-to-income ratio (0.36, 95% CI: 0.26–0.49). When comparing owners without a mortgage to those with a mortgage within the sample of lower-income households (Table 3 and Additional file 1: Table S5), the adjusted odds ratio was significant when including after-tax income (0.49, 95% CI: 0.29–0.80) but became non-significant when including after-housing income (0.76, 95% CI: 0.47–1.22) or housing-to-income ratio (0.87, 95% CI: 0.53–1.43). These results suggest that among lower-income households, differences in the burden of housing cost fully accounted for the disparities in food insecurity between owners with and without a mortgage, while differences in the burden of housing cost contributed minimally to the disparities between renters and both types of homeowner.

**Table 3:** Odds ratios of household food insecurity by homeownership status among lower-income households (*n* = 5547)

Independent variables	Unadjusted model	Covariates* + after-tax income	Covariates* + after-housing income	Covariates* + housing-to-income ratio
	OR (95%CI)	aOR (95%CI)	aOR (95%CI)	aOR (95%CI)
Homeownership status				
Renters	1.00	1.00	1.00	1.00
Owners with mortgage	**0.47 (0.36–0.60)**^a^	**0.53 (0.38–0.74)**^a^	0.41 (0.30–0.56)^a^	**0.36 (0.26–0.49)**^a^
Mortgage-free owners	**0.16 (0.10–0.24)**^b^	**0.26 (0.16–0.41)**^b^	0.31 (0.19–0.50)^a^	**0.31 (0.19–0.50)**^a^
After-tax income†	–	**0.93 (0.91–0.95)**	–	–
After-housing income†	–	–	**0.94 (0.92–0.96)**	–
Housing-to-income ratio†	–	–	–	**1.10 (1.05–1.14)**

aOR, adjusted odds ratios; OR, unadjusted odds ratios; CI, confidence interval

Note: The logistic regression models used sampling weights to obtain population-based OR and aOR; the 95% CI are calculated using bootstrapped standard errors estimated with 1000 bootstrap weights provided by Statistics Canada to account for the complex survey design. Odds ratios in bold are significantly different from 1.00 (*p values* < 0.05)

^a, b^ Based on comparisons of odds ratios from the same regression model (same column), odds ratios with different superscripts differ significantly from each other (*p values* < 0.05), while odds ratios with the same superscript do not differ (*p values* ≥ 0.05). See Additional file 1: Table S5 for the odds ratios for mortgage-free owners versus owners with a mortgage

* Covariates include household type, number of children < 18 years of age, presence of household member with disability, age of head of household, household education, main income source, region of residence and population centre size

† Odds ratios for $1000 increase in after-tax or after-housing income and for 5% increase in housing-to-income ratio

To examine the influence of housing asset on the relationship between homeownership status and food insecurity, homeowners with and without a mortgage were stratified based on the level of their housing asset. When adjusting for covariates and after-tax income, the odds ratio among owners with a mortgage and low housing asset compared to renters was non-significant (0.74, 95% CI: 0.44–1.27) (Table 4). In contrast, the adjusted odds of food insecurity were 50% lower among mortgage-free owners with low housing asset (0.50, 95% CI: 0.27–0.93), 52% lower among owners with a mortgage and higher housing asset (0.48, 95% CI: 0.36–0.64), and 82% lower among mortgage-free owners with higher housing asset (0.18, 95% CI: 0.11–0.27) compared to renters. Limiting the analyses to lower-income households had minimal impact on the adjusted odds ratios of owners with higher housing asset irrespective of whether they had a mortgage (0.50, 95% CI: 0.35–0.71) or not (0.18, 95% CI: 0.11–0.30) (Table 5). However, the adjusted odds ratios were non-significant for both types of owner with low housing asset when focusing on lower-income households. Overall, these results suggest that compared to renters, primarily homeowners with higher housing asset appeared to be less vulnerable to food insecurity, while owners with low housing asset seemed equally vulnerable.

**Table 4:** Odds ratios of household food insecurity by homeownership status and housing asset level* among households of all incomes (*n* = 10,815)

Independent variable	Unadjusted model	Covariates† + after-tax income
	OR (95% CI)	aOR (95% CI)
Homeownership status & housing asset level		
Renters	1.00	1.00
Owners with mortgage & low housing asset	0.85 (0.53–1.34)	0.74 (0.44–1.27)
Owners with mortgage & higher housing asset	**0.29 (0.23–0.36)**	**0.48 (0.36–0.64)**
Mortgage-free owners with low housing asset	**0.39 (0.21–0.75)**	**0.50 (0.27–0.93)**
Mortgage-free owners with higher housing asset	**0.07 (0.05–0.11)**	**0.18 (0.11–0.27)**

aOR, adjusted odds ratios; OR, unadjusted odds ratios; CI, confidence interval

Note: The logistic regression models used sampling weights to obtain population-based OR and aOR; the 95% CI are calculated using bootstrapped standard errors estimated with 1000 bootstrap weights provided by Statistics Canada to account for the complex survey design. Odds ratios in bold are significantly different from 1.00 (*p values* < 0.05)

* Low housing asset defined as home value ≤$120,000, representing the lowest decile of home value; higher housing asset defined as home value >$120,000

† Covariates include household type, number of children < 18 years of age, presence of household member with disability, age of head of household, household education, main income source, region of residence and population centre size

**Table 5:** Odds ratios of household food insecurity by homeownership status and housing asset level* among lower-income households (*n* = 5547)

Independent variable	Unadjusted model	Covariates† + after-tax income
	OR (95% CI)	aOR (95% CI)
Homeownership status & housing asset level		
Renters	1.00	1.00
Owners with mortgage & low housing asset	0.79 (0.51–1.23)	0.73 (0.42–1.27)
Owners with mortgage & higher housing asset	**0.42 (0.32–0.55)**	**0.50 (0.35–0.71)**
Mortgage-free owners with low housing asset	**0.41 (0.21–0.81)**	0.54 (0.26–1.13)
Mortgage-free owners with higher housing asset	**0.10 (0.06–0.15)**	**0.18 (0.11–0.30)**

aOR, adjusted odds ratios; OR, unadjusted odds ratios; CI, confidence interval

Note: The logistic regression models used sampling weights to obtain population-based OR and aOR; the 95% CI are calculated using bootstrapped standard errors estimated with 1000 bootstrap weights provided by Statistics Canada to account for the complex survey design. Odds ratios in bold are significantly different from 1.00 (*p values* < 0.05)

* Low housing asset defined as home value ≤$120,000, representing the lowest decile of home value; higher housing asset defined as home value >$120,000

† Covariates include household type, number of children < 18 years of age, presence of household member with disability, age of head of household, household education, main income source, region of residence and population centre size

The sensitivity analysis conducted among homeowners suggests that those with and without a mortgage had similar adjusted odds of food insecurity if they had low housing asset (Additional file 1: Table S6 and S7). In the whole sample, owners with a mortgage and higher housing asset had lower adjusted odds of food insecurity than those with a mortgage and low housing asset (Additional file 1: Table S6), but the difference in the adjusted odds ratios was non-significant in the lower-income sample (Additional file 1: Table S7). Owners with a mortgage and higher housing asset also had adjusted odds ratios similar to mortgage-free owners with low housing asset. Finally, mortgage-free owners with higher housing asset were least vulnerable to food insecurity, with adjusted odds ratios substantially smaller than those for all other categories of owner (Additional file 1: Table S6 and S7).

## Discussion

We found important disparities in food insecurity between households with different homeownership status and housing asset level. Our study adds to previous international research indicating that homeownership confers protection against food insecurity [12, 14, 16–26, 32] by showing that homeowners are not all equally protected and that their vulnerability appears to be related to both housing debt and the value of the housing asset. Consistent with earlier findings [12, 14, 16–26, 32], market renters were found to be substantially more vulnerable to food insecurity than most homeowners. It also appears that differences in income contribute more to the disparity in food insecurity between renters and homeowners than differences in housing cost burden.

This study expands current understandings of the economic drivers of food insecurity by suggesting that having a mortgage is a risk factor for food insecurity. Previous US research identified mortgage delinquency as a strong, independent predictor of food insecurity among mortgage holders [36], but to our knowledge, the relationship between having a mortgage and food insecurity had never been examined in affluent countries. Mortgage delinquency is rare in Canada, with national rates below 0.5% since the late 1990s [49], but our results indicate that the financial burden of paying off a mortgage contributes to food insecurity. Although outright homeownership is associated with the lowest vulnerability to food insecurity, the path towards outright ownership is not risk-free since most households must first service a substantial debt. In Canada, the federal government strongly influences households’ access to mortgages through lending regulations and mortgage insurance and securitization programs [28, 29, 49]. It also recently introduced a new initiative providing long-term, interest-free loans to low- and middle-income first-time homebuyers to reduce their monthly mortgage payments [50]. Research is needed to evaluate the impact of these programs on food insecurity among homeowners. However, our study and previous research [12, 14, 16–26, 32, 36] suggest that housing policy that promotes homebuyers’ access to an affordable mortgage may play a role in fostering household food security by containing the financial burden associated with having a mortgage and supporting the acquisition of housing asset.

It has long been hypothesized that the lower food insecurity risk associated with homeownership reflects the role of housing as an asset [16, 21, 22, 24]. We found that households owning a house with a value in the lowest decile had the highest food insecurity prevalence among homeowners and were as vulnerable to food insecurity as renters, suggesting that the protection conferred by homeownership may depend on the value of the home. While our results are consistent with previous studies showing an inverse, independent relationship between the value of the house owned and adverse health outcomes among older adults in Northern Ireland [51] and Spain [52], more research is needed to better understand how the value of the housing asset relates to food insecurity. Homeowners with low housing asset were overrepresented in the Atlantic region and Quebec, and predominantly lived in less populated areas (Additional file 1: Table S8). The higher propensity to own a low-value house in less populated areas likely reflects lower housing prices but may also indicate limited availability of rental accommodations within these areas. Thus, different policy actions may be required to promote household economic resilience in ways other than homeownership among those living in less populated areas. Future research should examine the relationship between homeownership, housing asset value and food insecurity within areas with lower housing prices to better understand the protective role of homeownership.

Although we found important disparities in food insecurity among homeowners, most of them are still considerably less vulnerable to food insecurity than renters, suggesting that housing policy may play an important role in mitigating food insecurity by supporting asset accumulation through homeownership. However, it is critical to recognize that stable and adequate income is often a prerequisite to access homeownership. Our results suggest that differences in income contribute significantly to the disparity in food insecurity between renters and homeowners. Housing policies played an instrumental role in increasing homeownership between the early 1990s and 2010s in Canada, but rates increased primarily among higher-income households while declining among lower-income households [29, 30, 49]. This indicates that housing policy promoting homeownership must be accompanied by other policy actions that support the economic resilience of lower-income renters. It is currently unclear to what extent government housing subsidy programs reduce vulnerability to food insecurity among low-income renting households in Canada, and thus, more research is needed to understand the role of these programs in mitigating food insecurity. However, the high levels of food insecurity [15, 39, 40] and the strong relationship between lower income and food insecurity [15] documented among households living in government-subsidized housing indicate that the economic support provided by government through these programs is insufficient to ensure food security among low-income renting households. Recent studies from Canada suggest that food insecurity is sensitive to policies increasing the amount and stability of incomes. More specifically, the risk of food insecurity has been shown to decline among low-income vulnerable households with the receipt of the universal old-age pension [10], the introduction of child benefits [11, 12], and improvements to social assistance benefits [13, 14]. With more than two thirds of food insecure households being renters in Canada [3], income-based interventions targeted to renting households could be effective at reducing food insecurity prevalence.

### Limitations

Although the analyses were population-based, they did not include a national sample of households since the 2010 SHS excluded households living in the territories and on First Nations reserves. It is unlikely, however, that the inclusion of households living in these areas would have substantially affected the results because they represent a small proportion of the overall Canadian population.

The analyses controlled for several potential confounders, but some that were identified in the literature could not be included due to a lack of data in the 2010 SHS; more specifically, data on Indigenous status and ethnicity were unavailable. It is unclear whether the inclusion of these confounders would have changed the interpretation of the results, since previous studies found that the heightened vulnerability of renters relative to homeowners persisted when controlling for a series of household characteristics including Indigenous status and ethnicity [12, 14, 16–20]. The relationship between renting versus owning a home and food insecurity found in this study and prior research [12, 14, 16–26, 32] may also be confounded by unobserved factors. Examples of household circumstance typically not measured in national surveys in Canada that could contribute to food insecurity and may be more common among renters than homeowners include precarious employment [53] and experiences of negative income shocks [23] or large expenditure shocks [54]. Thus, research using longitudinal datasets containing comprehensive information of sociodemographic characteristics and economic circumstances is required to better understand the factors contributing to the heightened vulnerability of renting households.

We lacked the data to account for mortgage size or delinquency and to test whether the association between owning a home of higher value and lower food insecurity was related to the value of the housing asset or to households having greater assets overall. Additional research using more detailed information on mortgage and different types of asset is needed. Although this study focused on Canada, the implications of our results are relevant to other country contexts. The adoption of housing policies promoting homeownership to build household’s asset is not unique to Canada [29, 35], and lower food insecurity risk among homeowners compared to renters has been documented in other affluent countries [22–26, 32].

## Conclusion

This study represents a novel examination of the intersection between homeownership status, housing debt, housing asset and vulnerability to food insecurity. The results suggest that housing policy may play a role in mitigating food insecurity by facilitating homeownership and reducing the financial vulnerabilities associated with mortgage debts. However, with most food insecure households being renters, it is critical to develop effective interventions targeted to lower-income renting households to strengthen their economic resilience.

## Supplementary information

**Additional file 1: Table S1.** Correlation matrix between total housing expenditure and its individual expenditure components among mortgage-free homeowners. **Table S2.** Correlation matrix between total housing expenditure and its individual expenditure components among homeowners with a mortgage. **Table S3.** Correlation matrix between total housing expenditure and its individual expenditure components among market renters. **Table S4.** Odds ratios of household food insecurity by homeownership status among households of all incomes (*n* = 10,815) - reference category set to owners with a mortgage. **Table S5.** Odds ratios of household food insecurity by homeownership status among lower-income households (*n* = 5547) - reference category set to owners with a mortgage. **Table S6.** Odds ratios of household food insecurity by homeownership status and housing asset level among homeowners of all incomes (*n* = 8360). **Table S7.** Odds ratios of household food insecurity by homeownership status and housing asset level among homeowners with lower incomes (*n* = 3690). **Table S8.** Geographical distribution of households with different homeownership status and housing asset level. **Fig. S1.** Prevalence of household food insecurity by deciles of estimated home value among homeowners

## Data Availability

The dataset analysed during the current study is not publicly available due to privacy constraints but is available through Statistics Canada’s Research Data Centres.

## Abbreviations

aOR: Adjusted odds ratios
CI: Confidence interval
EI: Employment Insurance
HFSSM: Household Food Security Survey Module
OR: Odds ratios
SHS: Survey of Household Spending
US: United States
