# Supplementary material for: Homeownership status and risk of food insecurity: examining the role of housing debt, housing expenditure and housing asset using a cross-sectional population-based survey of Canadian households
Source: Int J Equity Health. 2020 Jan 6;19:5. doi: 10.1186/s12939-019-1114-z (PMC6945525; doi:10.1186/s12939-019-1114-z)
Supplement: Supplementary file 1 — Additional file 1: Table S1. Correlation matrix between total housing expenditure and its individual expenditure components among mortgage-free homeowners. Table S2. Correlation matrix between total housing expenditure and its individual expenditure components among homeowners with a mortgage. Table S3. Correlation matrix between total housing expenditure and its individual expenditure components among market renters. Table S4. Odds ratios of household food insecurity by homeownership status among households of all incomes (n = 10,815) - reference category set to owners with a mortgage. Table S5. Odds ratios of household food insecurity by homeownership status among lower-income households (n = 5547) - reference category set to owners with a mortgage. Table S6. Odds ratios of household food insecurity by homeownership status and housing asset level among homeowners of all incomes (n = 8360). Table S7. Odds ratios of household food insecurity by homeownership status and housing asset level among homeowners with lower incomes (n = 3690). Table S8. Geographical distribution of households with different homeownership status and housing asset level. Fig. S1. Prevalence of household food insecurity by deciles of estimated home value among homeowners [file 12939_2019_1114_MOESM1_ESM.pdf]

Homeownership status and risk of food insecurity in Canada

ADDITIONAL FILE

Table S1 Correlation matrix between total housing expenditure and its individual expenditure components among mortgage-free homeowners

	Total housing	Utility	Rent	Tenant's insurance	Mortgage	Property tax	Condo fees	Owner's insurance	Mortgage insurance
Total housing	1								
Utility	0.4709*	1							
Rent	.	.	.						
Tenant's insurance	.	.	.	.					
Mortgage	.	.	.	.	.				
Property tax	0.7353*	0.2256*	.	.	.	1			
Condo fees	0.5097*	-0.2759*	.	.	.	0.0799*	1		
Owner's insurance	0.4723*	0.2245*	.	.	.	0.3647*	-0.0234*	1	
Mortgage insurance	.	.	.	.	.	.	.	.	.

* <0.05
All variables are continuous and represent expenditures adjusted for household size by dividing by the square root of household size. Blanks (.) indicate these expenditures were not reported by mortgage-free homeowners.

Table S2 Correlation matrix between total housing expenditure and its individual expenditure components among homeowners with a mortgage

	Total housing	Utility	Rent	Tenant's insurance	Mortgage	Property tax	Condo fees	Owner's insurance	Mortgage insurance
Total housing	1								
Utility	0.3411*	1							
Rent	.	.	.						
Tenant's insurance	.	.	.	.					
Mortgage	0.9595*	0.1725*	.	.	1				
Property tax	0.5191*	0.3036*	.	.	0.3672*	1			
Condo fees	0.1112*	-0.2868*	.	.	0.0306*	-0.0690*	1		
Owner's insurance	0.3077*	0.2972*	.	.	0.2196*	0.2900*	-0.1960*	1	
Mortgage insurance	0.1895*	0.0084*	.	.	0.1386*	-0.0082*	-0.0377*	0.0093*	1

* <0.05
All variables are continuous and represent expenditures adjusted for household size by dividing by the square root of household size. Blanks (.) indicate these expenditures were not reported by homeowners with a mortgage.

Table S3 Correlation matrix between total housing expenditure and its individual expenditure components among market renters

	Total housing	Utility	Rent	Tenant's insurance	Mortgage	Property tax	Condo fees	Owner's insurance	Mortgage insurance
Total housing	1								
Utility	0.1232*	1							
Rent	0.9791*	-0.0766*	1						
Tenant's insurance	0.2384*	0.0825*	0.1806*	1					
Mortgage	.	.	.	.	.				
Property tax	.	.	.	.	.	.			
Condo fees	.	.	.	.	.	.	.		
Owner's insurance	.	.	.	.	.	.	.	.	
Mortgage insurance	.	.	.	.	.	.	.	.	.

* <0.05

All variables are continuous and represent expenditures adjusted for household size by dividing by the square root of household size. Blanks (.) indicate these expenditures were not reported by market renters.

Figure S1 Prevalence of household food insecurity by deciles* of estimated home value among homeowners

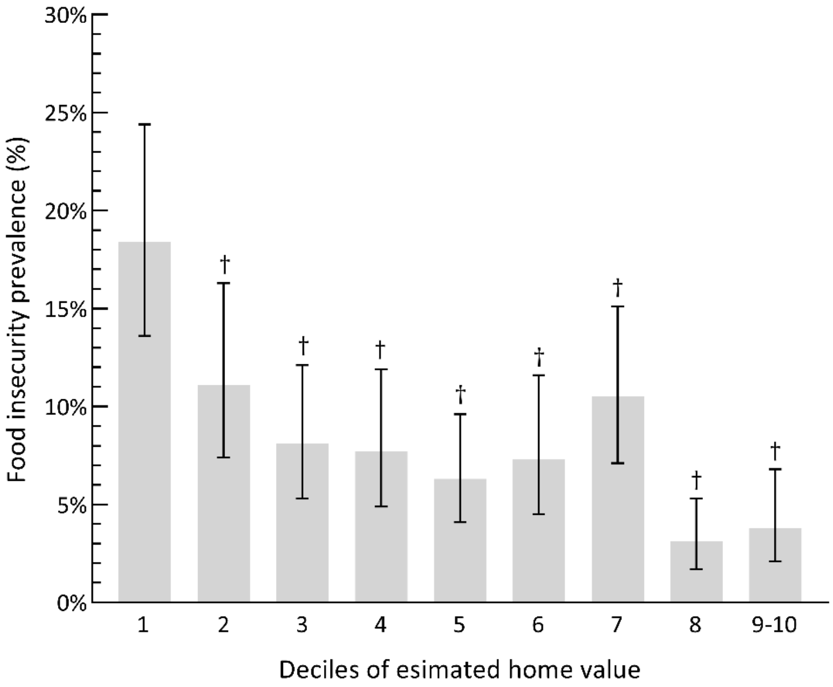

* Decile 9 and 10 were combined due to the few observations reporting food insecurity within each decile.

† Coefficient of variation greater than 16.6%, indicating that the estimate is associated with high sampling variability.

Table S4 Odds ratios of household food insecurity by homeownership status among households of all incomes (n=10815) – reference category set to owners with a mortgage

Homeownership status	Unadjusted model OR (95%CI)	Covariates* + after-tax income aOR (95%CI)	Covariates* + after-housing income aOR (95%CI)	Covariates* + housing-to-income ratio aOR (95%CI)
Renters	**3.04 (2.44-3.79)**	**1.94 (1.47-2.58)**	**2.33 (1.78-3.04)**	**3.19 (2.44-4.19)**
Owners with mortgage	1.00	1.00	1.00	1.00
Mortgage-free owners	**0.34 (0.24-0.49)**	**0.45 (0.30-0.68)**	**0.61 (0.41-0.90)**	0.81 (0.53-1.25)

aOR, adjusted odds ratios; OR, unadjusted odds ratios; CI, confidence interval.
Note: The logistic regression models used sampling weights to obtain population-based OR and aOR; the 95% CI are calculated using bootstrapped standard errors estimated with 1000 bootstrap weights provided by Statistics Canada to account for the complex survey design. Odds ratios in bold are significantly different from 1.00 (*p values* <0.05).
* Covariates include household type, number of children <18 years of age, presence of household member with disability, age of head of household, household education, main income source, region of residence and population centre size.

Table S5 Odds ratios of household food insecurity by homeownership status among lower-income households (n=5547) – reference category set to owners with a mortgage

Homeownership status	Unadjusted model OR (95%CI)	Covariates* + after-tax income aOR (95%CI)	Covariates* + after-housing income aOR (95%CI)	Covariates* + housing-to-income ratio aOR (95%CI)
Renters	**2.13 (1.65-2.76)**	**1.90 (1.35-2.66)**	**2.45 (1.78-3.38)**	**2.81 (2.02-3.90)**
Owners with mortgage	1.00	1.00	1.00	1.00
Mortgage-free owners	**0.34 (0.22-0.51)**	**0.49 (0.29-0.80)**	0.76 (0.47-1.22)	0.87 (0.53-1.43)

aOR, adjusted odds ratios; OR, unadjusted odds ratios; CI, confidence interval.
Note: The logistic regression models used sampling weights to obtain population-based OR and aOR; the 95% CI are calculated using bootstrapped standard errors estimated with 1000 bootstrap weights provided by Statistics Canada to account for the complex survey design. Odds ratios in bold are significantly different from 1.00 (*p values* <0.05).
* Covariates include household type, number of children <18 years of age, presence of household member with disability, age of head of household, household education, main income source, region of residence and population centre size.

Table S6 Odds ratios of household food insecurity by homeownership status and housing asset level* among homeowners of all incomes (n=8360†)

Homeownership status & housing asset level	Covariates‡ + after-tax income<br>aOR (95%CI)
Owners with mortgage & low housing asset	1.00
Owners with mortgage & higher housing asset	**0.58 (0.35-0.98)**[a]
Mortgage-free owners with low housing asset	0.60 (0.31-1.17)[a]
Mortgage-free owners with higher housing asset	**0.20 (0.11-0.38)**[b]

aOR, adjusted odds ratios; CI, confidence interval.
Note: The logistic regression models used sampling weights to obtain population-based OR and aOR; the 95% CI are calculated using bootstrapped standard errors estimated with 1000 bootstrap weights provided by Statistics Canada to account for the complex survey design. Odds ratios in bold are significantly different from 1.00 (*p values* <0.05).
[a, b] Based on comparisons of odds ratios from the same regression model, odds ratios with different superscripts differ significantly from each other (*p values* <0.05), while odds ratios with the same superscript do not differ (*p values* ≥0.05).
* Low housing asset defined as home value ≤$120,000, representing the lowest decile of home value; higher housing asset defined as home value >$120,000.
† Sample size rounded to nearest ten to respect Statistics Canada's confidentiality requirements.
‡ Covariates include age of head of household, household type, number of children <18 years of age, presence of household member with disability, household education, main income source, region of residence and population centre size.

Table S7 Odds ratios of household food insecurity by homeownership status and housing asset level* among homeowners with lower incomes (n=3690†)

Homeownership status & housing asset level	Covariates‡ + after-tax income<br>aOR (95%CI)
Owners with mortgage & low housing asset	1.00
Owners with mortgage & higher housing asset	0.62 (0.34-1.15)[a]
Mortgage-free owners with low housing asset	0.64 (0.29-1.45)[a]
Mortgage-free owners with higher housing asset	**0.21 (0.10-0.43)**[b]

aOR, adjusted odds ratios; CI, confidence interval.
Note: The logistic regression models used sampling weights to obtain population-based OR and aOR; the 95% CI are calculated using bootstrapped standard errors estimated with 1000 bootstrap weights provided by Statistics Canada to account for the complex survey design. Odds ratios in bold are significantly different from 1.00 (*p values* <0.05).
[a, b] Based on comparisons of odds ratios from the same regression model, odds ratios with different superscript differ significantly from each other (*p values* <0.05), while odds ratios with the same superscript do not differ (*p values* ≥0.05).
* Low housing asset defined as home value ≤$120,000, representing the lowest decile of home value; higher housing asset defined as home value >$120,000.
† Sample size rounded to nearest ten to respect Statistics Canada's confidentiality requirements.
‡ Covariates include age of head of household, household type, number of children <18 years of age, presence of household member with disability, household education, main income source, region of residence and population centre size.

Table S8 Geographical distribution of households with different homeownership status and housing asset level

	All	Renters	Owners with mortgage		Mortgage-free owners	
			low asset	higher asset	low asset	higher asset
Region of residence, %						
Atlantic	7.5	5.3	26.3	5.9	30.2	5.7
Quebec	26.1	35.6	37.6	23.3	30.8	19.1
Ontario	36.4	33.1	17.7	39.4	12.8	41.6
Prairies	17.4	14.1	14.1	19.0	18.7	18.5
British Columbia	12.6	11.9	4.2*	12.4	7.5	15.0
Population centre size, %						
Rural (<1000)	11.9	3.3	39.2	8.2	51.6	14.4
Small (1000 to <100,000)	20.9	18.4	34.5	19.8	36.0	20.4
Medium (100,000 to <500,000)	17.4	17.4	15.3*	18.3	8.7*	17.9
Large (≥500,000)	49.9	60.8	11.0*	53.7	3.7*	47.4

Note: column percentages may not add to 100% due to rounding.
* Coefficient of variation greater than 16.6%, indicating that the estimate is associated with high sampling variability.
